# Drug-Induced Liver Injury: Biomarkers, Requirements, Candidates, and Validation

**DOI:** 10.3389/fphar.2019.01482

**Published:** 2019-12-11

**Authors:** Lucy Meunier, Dominique Larrey

**Affiliations:** Liver and Transplantation Unit, Montpellier School of Medicine and IRB-INSERM-1183, Montpellier, France

**Keywords:** biomarkers, drug-induced liver injury, hepatotoxicity, RUCAM, novel candidate

## Abstract

The hepatotoxicity of drugs is the main cause of drug withdrawal from the pharmaceutical market and interruption of the development of new molecules. Biomarkers are useful in several situations. In case of suspected drug-induced liver injury (DILI), biomarkers can be used to confirm liver damage, its severity, prognosis, confirm drug causality, or define the type of DILI. In this review, we will first present the currently used biomarkers and candidate biomarkers for the future. The current biomarkers are certainly very helpful including with the assistance of diagnostic method such the Roussel Uclaf Causality Assessment Method, but provide a limited information for the early detection of liver injury, the role of specific drug and the prediction of DILI. Some biomarkers are promising but they are not yet available for routine use. Studies are still needed to confirm their interest, particularly in comparison to Roussel Uclaf Causality Assessment Method.

## Introduction

The hepatotoxicity of drugs is the main cause of drug withdrawal from the pharmaceutical market and interruption of the development of new molecules. Its incidence in the general population varies from 2.4/100,000 to 19/100,000 depending on whether the studies are retrospective or prospective (Sgro et al., 2002; Björnsson et al., 2013; Larrey et al., 2017; Andrade et al., 2019). Liver damage is so diverse that it reproduces almost all non-iatrogenic liver diseases (Larrey et al., 2017; Andrade et al., 2019).

More than 1300 “classic” medicines are currently listed, to which the role of medicinal plants, food supplements and chemicals is increasingly added (Larrey et al., 2017; Andrade et al., 2019). Acute hepatitis is by far the most common manifestation, accounting for more than 90% of drug-induced liver disease (Larrey et al., 2017; Andrade et al., 2019).

There are two types of drug-induced liver injury (DILI). Direct, intrinsic toxicity, which is generally dose-dependent, with a fairly known mechanism making the diagnosis easier. The most well-known direct toxicity model is the hepatotoxicity of paracetamol. The second type of toxicity is idiosyncratic. It is rare, unpredictable and occurs at therapeutic doses in recommended situation of prescription. The large majority of drug-induced liver injuries occurs in an idiosyncratic manner. It is particularly in this situation that biomarkers are very useful (Larrey et al., 2017; Andrade et al., 2019).

According to a definition proposed in 1993, a biomarker is a sensitive laboratory test that is specific enough to confirm the drug-related nature of a liver injury. Ideally, a hepatotoxicity biomarker should not only be the signature of a liver lesion, but should also identify the xenobiotic involved or at least one class of chemical entities (Andrade et al., 2019).

Biomarkers are useful in several situations. In case of suspected DILI, biomarkers can be used to confirm liver damage, its severity, prognosis, confirm drug causality, or define the type of DILI (Andrade et al., 2019).

In this review, we will first present the currently used biomarkers and candidate biomarkers for the future.

## Currently Used Biomarkers

### Diagnostic Biomarkers

**Figure 1:**
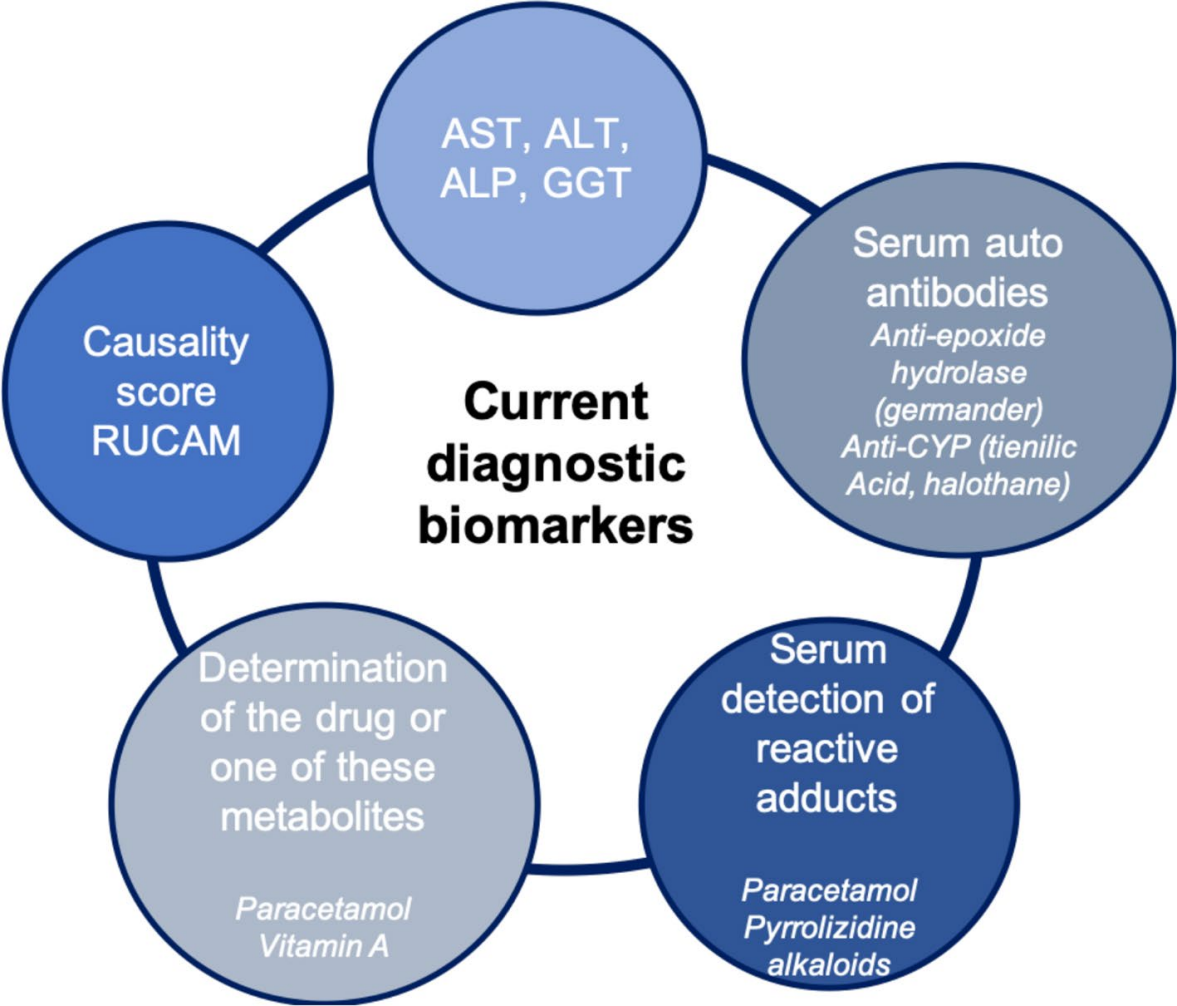
Current diagnostic biomarkers.

Currently, there are few biomarkers for DILI that are useful for early detection, monitoring, or for diagnosis purposes (**Figure 1**). Traditionally, in clinical practice, the biomarkers used to detect liver injury measure either an alteration in the normal liver function, changes in tissue and cell integrity, detected by serum levels of alanine aminotransferase (ALT), aspartate aminotransferase, alkaline phosphatase (ALP), and gamma-glutamyl transferase (Aithal et al., 2011; Andrade et al., 2019). The usefulness of serum bilirubin as diagnostic marker depends of its level. When total bilirubin is lower than 40 µmol/L without value of conjugated bilirubin, it may be confusing with increased unconjugated bilirubin as observed with Gilbert disease. However, when its value is largely above this level or when the increase is associated with increased conjugated bilirubin, it is a diagnostic sign of liver injury. Nevertheless, the use of bilirubin is more a criteria of severity (see the *Severity Biomarkers* section). The EASL DILI guidelines (Andrade et al., 2019) proposed the following case definitions for DILI include one of the following thresholds: 

- Serum ALT elevation ≥5 times the upper limit of normal value (ULN)
- Serum ALP ≥2 × ULN (particularly with accompanying elevations of gamma-glutamyl transferase in the absence of known bone pathology driving the rise in ALP level)
  or
- The combination of ALT ≥3 × ULN elevation with simultaneous elevation of total bilirubin concentration exceeding 2 × ULN (2)

Although these traditional biomarkers can reflect hepatic lesions, being useful for the diagnosis of severe DILI, they have many limitations that in practice do not make them ideal biomarkers. Increased serum level of ALT and aspartate aminotransferase is commonly used as a biomarker of hepatocellular injury, though its elevation can also be typical of muscle and cardiac damage, respectively, demonstrating its poor specificity. In addition, these biomarkers do not allow to distinguish DILI from other etiologies of liver injury, or identify its specific causative agent. The levels of liver enzymes also have a poor correlation with histological patterns and lesion severity (Devarbhavi, 2012).

Thus, currently, the diagnosis of DILI is mainly based on chronological criteria, clinical criteria, and the elimination of other competitive causes. In the absence of specificity in the majority of cases, it is often a diagnosis of elimination (; Fontana et al., 2010; Larrey et al., 2017). The determination of causality can find help by using methods based on scores ascribed to the relevant parameters. Several causality assessment methods (CAM) have been developed based on scores. The main one is the Roussel Uclaf Causality Assessment Method (RUCAM).There is also other method of causality assessment such the American Drug-Induced Liver Injury Network system which is not based on scores but on a probability estimation of causality (Fontana et al., 2009). The most commonly used CAM is the RUCAM which has recently been updated (Danan and Teschke, 2015).

#### Determination of the Drug or One of These Metabolites

A prototype is paracetamol, whose toxicity mechanism is direct, predictable and dose dependent. Plasma paracetamol concentration is directly correlated with hepatic toxicity (>200 µg/L 4 h or >100 µg/L 8 h after ingestion) (; Andrade et al., 2019).

#### Specific Autoantibodies

The hepatotoxicity of some drugs is associated with the presence of specific antibodies. They combine very good specificity and sensitivity and are a very good diagnostic marker. This is the case for anti-mitochondrial antibodies type 6 with isoniazid, anti-LKM2 or anti-cytochrome 2C9 with tienilic acid, anti-cytochrome 1A2 with dihydralazine, anti-cytochrome 3A with anti-epileptics, and anti-cytochrome 2E1 with halothane (Larrey et al., 2017).

Another interesting example is an anti-epoxide hydrolase antibody, a specific marker for hepatotoxicity of germander (*Teucrium chamaedrys*). This medicinal plant, initially used as an antipyretic and analgesic for abdominal pain, obtained a marketing authorization in 1986 as an aid to weight loss (Larrey and Faure, 2011; Teschke et al., 2016). In a few months, more than 30 cases of drug-related liver injury were collected by pharmacovigilance center, including fulminant hepatitis. It has therefore been withdrawn from the market and its free sale is now prohibited. The mechanism of hepatotoxicity was then demonstrated: the oxidation of Germander by CYP 3A leads to the formation of reactive metabolites that are the target in the blood of anti-epoxide hydrolase antibodies (Larrey and Faure, 2011; Teschke et al., 2016).

#### Serum Detection of Reactive Adducts

Another example of a biomarker based on one of the main toxicity mechanisms is the formation of toxic reactive metabolites from the drug (Larrey et al., 2017). This toxic metabolite can bind irreversibly to various organelles and molecular structures, including proteins. The reactive protein-reactive metabolite complex forms an adduct that can be detected in the blood. The detection of a paracetamol metabolite-protein adduct in blood of patients with paracetamol-induced liver injury has been demonstrated but this method of diagnosis but is not entered in the clinical practice and finally not necessary in most of situations (James et al., 2009). Another recent example is the detection in the blood or specific urine of a toxic metabolite formed from pyrrolizidine alkaloids (Larrey and Faure, 2011). At the origin of the implementation of this biomarker, “Tusanqi,” a traditional Chinese preparation used for analgesic purposes, normally without risk but which has led to a series of 50 cases of sinusoidal obstruction syndrome (Teschke et al., 2016). Toxicity has been linked to confusion between two plants during the manufacture of this preparation, the harmless *Sedum aizoon* has unfortunately been replaced by *Gynura segetum* containing toxic alkaloids (Teschke et al., 2016). A biomarker of pyrrolizidine alkaloids was introduced, initially tested in rats and then in a patient with sinusoidal obstruction syndrome but with a favorable evolution and allowed the diagnosis of certainty with a specificity of 95.8% and a sensitivity of 100%. The level of adducts of reactive pyrrole-protein reactive metabolites decreases rapidly during the first 40 days but remains detectable in the blood for about 300 days (Larrey and Faure, 2011; Lin et al., 2011; Teschke et al., 2016).

### Severity Biomarkers

The severity of the disease varies greatly, from a simple increase in transaminases to fatal fulminant hepatitis (Larrey et al., 2017; Andrade et al., 2019). Drugs are the leading cause of fulminant hepatitis. The assessment of the severity of DILI is based on a combination of biological and clinical criteria [increased bilirubin and alteration of blood clotting markers (proaccelerin, international normalized ratio, prothrombin time) and decrease of serum albumin] (Andrade et al., 2019). During the development of a new drug, it is important to be able to predict the occurrence of severe hepatotoxicity. Many years ago, Hyman Zimmerman demonstrated that, when acute cytolytic DILI becomes complicated by jaundice, the risk of severe liver failure was about 10% (Mayoral et al., 1999). The Food and Drug Administration has extrapolated the “Hy’s law rule” characterized by the combination of ALT > 3 × ULN and total bilirubinemia > 2 × ULN as an alert signal for the risk of severe hepatotoxicity in acute cytolytic attacks after eliminating a non-drug cause (Mayoral et al., 1999). This rule is now systematically applied in clinical trials involving drugs in development. In addition, a graphical representation of this combination has been created, the “eDISH plot” (Evaluation of Drug-Induced Serious Hepatotoxicity), to facilitate the detection of the risk of ser hepatotoxicity events occurring in therapeutic trials (Mayoral et al., 1999; Watkins et al., 2011).

To summarize, the current biomarkers are certainly very helpful including with the assistance of diagnostic method such the RUCAM, but provide a limited information for the early detection of liver injury, the role of specific drug and the prediction of DILI.

## Novel Candidate Biomarkers

The different candidate biomarkers may be classified according to their usefulness (diagnosis or prognosis) and their comparison to RUCAM as shown in the **Table 1**.

**Table 1:** New biomarkers in drug-induced liver injury (DILI): diagnostic and prognostic value.

Biomarkers	Mechanism	Diagnostic value	Prognostic value	RUCAM evaluation
Mir 122 et 192 (Teschke et al., 2016; Howell et al., 2018)	Noncoding RNAs involved in regulation of cellular processes	X	X	No
Cytokeratin 18 (Thulin et al., 2014; Church and Watkins, 2017; Kullak-Ublick et al., 2017; Church et al., 2019)	Cytoskeleton protein	X	X	No
GLDH (Harrill et al., 2012; Schomaker et al., 2013; Church et al., 2019)	Mitochondrial enzyme	X		No
MCSFR1 (Kullak-Ublick et al., 2017; Barnhill et al., 2018; Church et al., 2019).	Marker of immune activation		X	No
Bile acids		X		No
Pharmacogenetics			X	No
GST-alfa (Church et al., 2019)	Intracellular cytosolic enzyme	X	X	No
MDH (Schomaker et al., 2013)	Constitutive enzyme in the citric acid cycle	X		No
Osteopontin (Church et al., 2019)	Small integrin-binding N-linked glycoprotein	X	X	No
MH cells (Benesic et al., 2012; Benesic et al., 2016; Kullak-Ublick et al., 2017; Benesic et al., 2018)	MH cells from DILI patients used for *in vitro* toxicity testing of culprit compound	X		Yes
Metabolomic		X		No

X, yes.

### Micro RNA 122

MicroRNAs (miRNAs) are short RNAs that regulate gene expression after transcription, controlling translation of proteins and playing a major role in the regulation of cellular processes.

The circulating microRNAs, miR-122 and miR-192 are hepato-specific. MicroRNA 122 appears to be an early marker of liver damage (viral, alcohol, or toxic). In paracetamol intoxications, microRNA 122 is more sensitive and is increased earlier than the increase in transaminases. In case of muscle damage, there is no increase in microRNA 122 in the blood, unlike ALT. It is therefore more specific in this situation.

Howell et al. recently published a review article highlighting how miRNAs, such as miRNA-122, have the potential to provide both greater sensitivity and specificity in predicting, monitoring, and classifying DILI (Howell et al., 2018).

### Cytokeratin 18

It is a cytoskeleton protein that is very abundant in the liver but not specific. It is released in case of hepatocyte necrosis. Its increase is early in case of hepatic toxicity, before that of ALT. It could also be a prognostic marker of hepatic damage because it is more increased in patients who die or transplant after paracetamol overdose compared to patients who have spontaneously recovered (Thulin et al., 2014; Church and Watkins, 2017; Kullak-Ublick et al., 2017; Church et al., 2019).

### GLDH (Glutamate Dehydrogenase)

GLDH is a protein that is embedded in the matrix of the mitochondria that is involved in oxidative deamination of glutamate. The liver is abundant in matrix-rich mitochondria and is therefore highly enriched for GLDH while much smaller amounts are present in the kidneys and brain. Tissues abundant in cristae-rich mitochondria, such as skeletal muscle, do not have appreciable amounts of GLDH, giving this biomarker an advantage over ALT in terms of liver specificity. A multicenter study comparing patients with DILI, healthy volunteers, and patients who have taken potentially toxic drugs but without side effects tested the performance of several biomarkers. GLDH was more useful than miR-122 in identifying DILI patients (Church et al., 2019).

### MCSFR1 (Macrophage Colony-Stimulating Factor Receptor 1)

It is another marker of immune system activation. It is greatly increased in cases of idiosyncratic hepatotoxicity. For example, it increases in the case of idiosyncratic hepatotoxicity secondary to flupirtin and not in the case of paracetamol toxicity (direct toxicity), while transaminases are increased in both cases (Kullak-Ublick et al., 2017; Barnhill et al., 2018; Church et al., 2019).

MCSFR1 and osteopontin are higher in patients with hepatotoxicity and severity criteria according to the “Hy’s law” rule compared to patients without severity criteria (Church et al., 2019).

### Bile Acids

The increase in certain serum bile acids (glycochenodeoxycholic acid, taurochenodeoxycholic acid, taurochenodeoxycholic acid, taurocholic acid) has been shown in some cases of hepatotoxicity (flupirtin) even in the absence of biological cholestasis. They are associated with the severity of liver injury because they are more increased in patients with fulminant hepatitis (Luo et al., 2014).

Recently, in a multicenter study, Church et al. aimed to study the performance characteristics of previously studied biomarkers using several cohorts (healthy volunteers, patients taking potentially hepatotoxic medications without adverse effect, DILI patient). The authors noted that GLDH correlated better with ALT than miRNA-122 and that keratin 18, osteopontin, and MCSFR levels correlated best with liver-associated death/transplant within 6 months (Church et al., 2019).

### Pharmacogenetics

For a long time, a link between idiosyncratic, rare, and unpredictable drug liver injury and genetic factors has been increasingly demonstrated at the population level. However, few robust combinations have been found between pharmacogenetics and specific drugs (Aithal et al., 2004; Andrade et al., 2009; Chalasani and Björnsson, 2010; Andrade et al., 2019).

To date, genetic studies in this field have been based on the hypothesis of the candidate gene. More recently, genome-wide studies have been conducted in patients with DILI (Nicoletti et al., 2017; Urban et al., 2017; Cirulli et al., 2019). This has allowed the development of a new toxicity model for idiosyncratic drug liver injury involving drug-specific metabolic pathways that generate reactive metabolites and common signaling pathways leading to cellular stress and necrosis (directly or via immune reactions). The identified genetic variants are presented according to their role in the metabolism of the drug.

### Phase 1 Metabolism Enzymes

The formation of reactive metabolites by cytochrome P450 plays a key role in the pathophysiology of DILI. Many studies have focused on the different variants of the cytochrome and their involvement, but a robust link has only been established in rare cases.

### Phase 2 Detoxification Enzymes

N acetyltransferase 2 (NAT2) is involved in DILI. There are several variants with different acetylation activity. Low acetylation capacity is associated with an increased risk of hepatotoxicity due to sulfonamides and isoniazid.

Glutathione transferase T1 and M1 is a cytosolic enzyme that protects the cell from oxidative stress, a reduction in its activity (genotype GST T1 and M1 null) is associated with a higher risk of hepatotoxicity of antibiotics and NSAIDs for example.

Manganese superoxide-dismutase (MnSOD) is a mitochondrial enzyme also involved in protecting the cell from oxidative stress. The increased activity of this enzyme is associated with an increased risk of hepatotoxicity. The mechanism is not completely elucidated, perhaps through the increased production of hydrogen peroxide.

Although glucuronoconjugation is a detoxification mechanism, it can sometimes lead to the production of reactive toxic metabolites. This is the case, for example, of diclofenac which, when glucuronoconjugated by UGT2B7, produces acyl glucuronides that may be toxic to hepatocytes. Diclofenac is mainly metabolized by CYP2C9 but the presence of at least one UGT2B7*2 variant allele is associated with a high risk of hepatotoxicity (Aithal et al., 2004; Andrade et al., 2009).

### Hepatobiliary Transporters

The hepatic detoxification of xenobiotics is done *via* their conjugation with glutathione, sulfates, or glucuronates. These conjugated metabolites are then transported by hepatobiliary transporters outside the hepatocyte, this step constitutes a target of hepatotoxicity. The excretion of xenobiotics in bile involves the MRP family carriers: MDR1 (ABCB1), MDR3 (ABCB4), MRP2 (ABCC2), and BSEP (ABCB11). Cholestatic hepatitis secondary to sulindac, flucloxaciline, terbinafine, and bosentan are associated with inhibition of ductal BSEP. Patients with mutations in the genes encoding BSEP and MDR3 are three times more likely to develop cholestatic hepatitis secondary to oral contraception, certain antibiotics, proton pump inhibitors, and psychotropic drugs.

### Major Histocompatibility Complex

There is an established association between certain HLA polymorphisms and hepatic or non-hepatic drug side effects (Barnhill et al., 2018). One of the first associations highlighted is that between HLAB1*1501-DRB5*010101-DQB1*0602 and amoxicillin clavulanic acid, concerns 57% of patients with liver injury with amoxicillin clavulanic acid versus 12% of healthy patients (Andrade et al., 2009).

The HLA genotype is a factor influencing the phenotype of liver injury. For example, Andrade et al. found that cholestatic or mixed hepatitis had more frequent haplotype HLA-DRB1*15 and HLA-DQB1*06 and less frequent haplotype DRB1*07 and DQB1*02 than cytolytic hepatitis (Andrade et al., 2009).

In addition, HLA genotype may be useful for the diagnosis of drug hepatotoxicity. Indeed, recently ximelagatran, an oral anticoagulant, has been withdrawn from the market due to its hepatotoxicity. Genome analysis revealed a haplotype HLA DRB1*07 [odds ratio (OR) 4.41] and DQA1*02 (OR 4.41) in patients with DILI. Similarly, a very strong association between the hepatotoxicity of flucloxacillin and HLA-B*5701 (OR 80.6) has been demonstrated (Daly et al., 2009). Recently, an association between HLA-B*35:01 and *Polygonum multiflorum* induced DILI (Chinese herbal medicine) (Li et al., 2019).

The HLA-B*35:02 genotype is a useful diagnostic test for liver injury secondary to minocycline because it differentiates idiopathic autoimmune hepatitis from drug-induced autoimmune hepatitis while serological markers (anti-nuclear Ac and smooth muscle) may be present in both cases (Urban et al., 2017). In this study Urban et al. use RUCAM as the primary causality scoring method to confirm DILI (Urban et al., 2017).

To date the only drug in which HLA genotyping is mandated before receiving the drug is abacavir, a nucleoside reverse transcriptase inhibitor used in HIV treatment (Mallal et al., 2008). The PREDICT-1 trial demonstrated that by screening for this particular HLA marker, the incidence of abacavir-hypersensitivity reactions was decreased from 2.7% to 0% (Mallal et al., 2008).

The positive predictive value of the HLA markers is low whereas their negative predictive value is quite high. Thus, these HLA haplotypes may prove to be more beneficial in the diagnosis of DILI by being able to rule out a specific drug as the cause of the liver injury, however this is ultimately dependent on the frequency of DILI by a specific drug in a specific population (Clare et al., 2017).

In a multi-center study, Ocete-Hita et al. selected children with high suspicion of DILI diagnosed using the RUCAM causality scale and compared their HLA genotypes to those of a control group. They found that the children with the HLA-DQA0102 and HLA-DR*12 markers had more frequently DILI, suggesting their possible use as a genetic risk factor. In contrast, HLA-C0401 and HLA-DQB0603 markers were more likely to be seen in the control patients without evidence of DILI, suggesting a possible hepato-protective effect with these HLA haplotypes (Ocete-Hita et al., 2017).

### Deregulation of Cytokines

Deregulation of cytokine production may be involved in the pathogenesis of DILI. Variants of interleukin 10 and 4 are associated with hepatotoxicity secondary to diclofenac (Bonkovsky et al., 2018). A study including 127 patients with acute hepatitis from the US Drug-Induced Liver Injury Network registry established a model including albumin < 28 g/L and Regulation upon activation, normal T expressed and secreted (RANTES) below the median value to predict premature death or transplantation in 81% of cases (Bonkovsky et al., 2018).

### Metabolomics

Metabolomic study have emerged as new approaches to the discovery of more sensitive and specific DILI biomarkers (Araújo et al., 2017). For example, monitoring metabolite levels in urine, has proven to be an important strategy in hepatotoxicity studies, used to screen potential earlier diagnostic and prognostic biomarkers (Beger et al., 2010; Zhang et al., 2012a; Zhang et al., 2012b).

### Proteomics

Mikus et al. performed a study using a proteomics approach to screen 4594 antibodies and 1196 samples from 241 individuals ranging from healthy volunteers receiving acetaminophen or heparins, patients with human immunodeficiency virus or tuberculosis receiving treatment, and known DILI cases. They observed elevated levels of cadherin 5 (CDH5) and fatty acid-binding protein 1 (FABP1) in DILI. Furthermore, in longitudinal cohorts, CDH5 seemed to be elevated at baseline in DILI cases and FABP1 seemed to respond more rapidly to treatment initiation than ALT. Thus, the authors proposed CHD5 as a susceptibility biomarker and FABP1 as a potential biomarker with better kinetics than ALT (Mikus et al., 2017).

### Monocyte Derived Hepatocyte Like

A German team has set up a diagnostic test for idiosyncratic drug liver injury. This is an *in vitro* method that allows a blood sample to be taken to culture MH (monocyte derived hepatocyte like) and to test the drugs possibly involved (Benesic et al., 2012; Benesic et al., 2016; Benesic et al., 2018). They investigate whether peripheral human monocytes after cultivation according to a novel protocol.

(MH cells) can serve as an *in vitro* model for hepatocyte metabolism. Enzyme activities, synthesis parameters (coagulation factor VII and urea), and cytochrome (CY) P450 activities and induction were investigated. Furthermore, MH cells were compared with primary human hepatocytes from the same donor. Using this protocol, the authors claimed that this system generate cell exhibiting hepatocyte like properties. Correlations between some drugs and cell injury has been found. A limit of the method that test as performed unblinded. This method has not be compared to RUCAM a part for a single case (Krisai et al., 2019).

### *In Silico* Modeling of DILI

Quantitative systems pharmacology is an “*in silico*” model that incorporates both liver physiology and *in vitro* experimental evidence to predict potential hepatotoxicity, in addition to the events that lead to liver injury (Woodhead et al., 2017).

### Dilisym

DILIsym^®^, a platform created by the DILIsym Initiative, a public-private partnership, that attempts to predict and define hepatotoxicity during drug development phases, including clinical trials.

This mathematical software integrates many parameters (different liver cells, intracellular biochemical systems, drug distribution and metabolism, inter-individual variability, etc.) to predict the risks of hepatotoxicity of drugs under development. For example, it has been successfully used to explore divergent toxicological responses for tolcapone and entecapone, two drugs used in clinical studies of Parkinson’s disease in humans (Longo et al., 2016).

For another example of the potential utility of DILIsym^®^, Longo et al. specifically studied cimaglermin alfa. This drug being developed for heart failure therapy and was prematurely stopped during the early phases of its clinical trial due to rise of both ALT and total bilirubin (fulfilling Hy’s Law) in two patients. These researchers incorporated DILIsym^®^ software and calculated an apoptotic index for the drug and found that apoptosis (as opposed to necrosis) was the primary mechanism for the cell death, and that the actual percentage of hepatocyte death was significantly lower than previously thought (Longo et al., 2017).

## Discussion

Idiosyncratic DILI is a challenging disease when it comes to clearly establishing its diagnosis. The newly proposed biomarkers and methods for the early detection of DILI are promising but raised the crucial question of validity, specificity, and sensitivity.

A first challenge is the understanding of the mechanism(s) of DILI. A recent review of DILI mechanisms (Uetrecht, 2019) showed that there is a large amount of evidence to support the hypothesis that most IDILI is immune mediated; however, there may very well be exceptions. Importantly, it is dangerous to extrapolate from the results of *in vitro* studies to infer the mechanism of IDILI without confirming that the *in vitro* results are consistent with clinical observations. For example, BSEP inhibition may be involved in the mechanism of some cases of idiosyncrasic DILI, but there is insufficient clinical data to provide confidence that this is a common mechanism, or to determine exactly what role BSEP inhibition plays in the overall mechanism (Uetrecht, 2019). The contributive role of inflammation is an attractive mechanism for the initiation of an immune response that can lead to idiosyncrasic DILI, but it is too early to know if this is a general mechanism. The fact that there is an association between the idiosyncratic DILI caused by several drugs and specific HLA genotypes suggests that the injury is caused by an adaptive immune response, and that is consistent with the impaired immune tolerance model and liver histology.

Another challenging point is that the validity of new biomarkers requires comparison with already validated biomarkers and robust CAMs. To date, the mostly recognized CAM is the updated RUCAM published in 2016. In this issue of the journal, an article is focused on the current analysis of the publications reporting on more than 46,000 cases which shows that RUCAM performs well provided that the RUCAM is correctly used. This article stresses the importance to get a very good collection of the data to allow a precise and performing causality assessment (Teschke, 2019).

## Conclusion

Drug-induced liver injuries are common, but diagnosis and prognosis are often difficult to establish, especially for idiosyncratic reactions.

The diagnosis is essentially based on clinical and biological criteria and chronology as the most commonly used score called RUCAM.

The causality score has limits and the great challenge that remains is to differentiate DILI from liver injury due to other causes and to predict the outcome.

In recent years, many teams have tried to set up biomarkers to address these issues. Collaborative efforts are ongoing in this area (Pro Euro DILI Registry Network, IMI.

SAFE-T, TransBioLine, and the Predictive Safety Testing Consortium). Some biomarkers are promising but they are not yet available for routine use. Studies are still needed to confirm their interest, particularly in comparison to RUCAM.

## Author Contributions

LM and DL: manuscript preparation, critique, and review. All authors approved the manuscript submitted.

## Conflict of Interest

The authors declare that the research was conducted in the absence of any commercial or financial relationships that could be construed as a potential conflict of interest.

## Abbreviations

DILI, drug-induced liver injury; ALT, alanine aminotransferase; AST, aspartate aminotransferase; ALP, alkaline phosphatase; GGT, gamma-glutamyl transferase; ULN, upper limit of normal value; CAM, causality assessment methods; RUCAM, Roussel Uclaf Causality Assessment Method; DILIN, Drug-Induced Liver Injury Network; PT, prothrombin time; miRNAs, microRNAs; FDA, Food and Drug Administration; MCSFR1, macrophage colony-stimulating factor receptor 1; NAT2, N acetyltransferase 2; MnSOD, manganese superoxide-dismutase; RANTES, Regulation upon activation normal T expressed and secreted; CDH5, cadherin 5; FABP1, fatty acid-binding protein 1; MH, monocyte derived hepatocyte like.
